# The Mobilization and Recruitment of C-Kit+ Cells Contribute to Wound Healing after Surgery

**DOI:** 10.1371/journal.pone.0048052

**Published:** 2012-11-14

**Authors:** Yoshihiro Takemoto, Tao-Sheng Li, Masayuki Kubo, Mako Ohshima, Hiroshi Kurazumi, Kazuhiro Ueda, Tadahiko Enoki, Tomoaki Murata, Kimikazu Hamano

**Affiliations:** 1 Department of Surgery and Clinical Science, Yamaguchi University Graduate School of Medicine, Ube, Yamaguchi, Japan; 2 Department of Stem Cell Biology, Life Science and Radiation Research, Nagasaki University Graduate School of Biomedical Science, Sakamoto, Nagasaki, Japan; 3 Institute of Laboratory Animals, Yamaguchi University, Ube, Yamaguchi, Japan; National Institutes of Health, United States of America

## Abstract

Delayed wound healing is a serious clinical problem in patients after surgery. A recent study has demonstrated that bone marrow-derived c-kit-positive (c-kit^+^) cells play important roles in repairing and regenerating various tissues and organs. To examine the hypothesis that surgical injury induces the mobilization and recruitment of c-kit+ cells to accelerate wound healing. Mice were subjected to a left pneumonectomy. The mobilization of c-kit+ cells was monitored after surgery. Using green fluorescent protein (GFP^+^) bone marrow-transplanted chimera mice, we investigated further whether the mobilized c-kit+ cells were recruited to effect wound healing in a skin puncture model. The group with left pneumonectomies increased the c-kit^+^ and CD34^+^ stem cells in peripheral blood 24 h after surgery. At 3 days after surgery, the skin wound size was observed to be significantly smaller, and the number of bone marrow-derived GFP^+^ cells and GFP^+^/c-kit+ cells in the wound tissue was significantly greater in mice that had received pneumonectomies, as compared with those that had received a sham operation. Furthermore, some of these GFP^+^ cells were positively expressed specific markers of macrophages (F4/80), endothelial cells (CD31), and myofibroblasts (αSMA). The administration of AMD3100, an antagonist of a stromal-cell derived factor (SDF)-1/CXCR4 signaling pathway, reduced the number of GFP^+^ cells in wound tissue and completely negated the accelerated wound healing. Surgical injury induces the mobilization and recruitment of c-kit+ cells to contribute to wound healing. Regulating c-kit+ cells may provide a new approach that accelerates wound healing after surgery.

## Introduction

Delayed wound healing continues to be a serious clinical problem after surgery because it increases the risk of surgical site infections [1], [2], extends postoperative hospitalizations [3], and increases medical expenses [4]–[7]. Although many countermeasures exist, delayed wound healing and wound infection are still common occurrences, especially in patients with an advanced age, diabetes mellitus, chronic renal failure, or other systemic diseases.

Wound healing involves complex processes, and many factors may contribute to delay these processes. Wound healing of the skin is a dynamic process involving fibroplasia, angiogenesis and re-epithelialization. Wound inflammation is central to the formation of new tissue. It is generally agreed that wound macrophages play an important role in wound healing [8], and dermal fibroblasts/myofibroblasts involved in wound healing are thought to originate from the resident fibroblast progenitors. [9] Recent studies have demonstrated that stem/progenitor cells play important roles in promoting the development of new vessels [10]–[13], one of the critical processes during early wound healing, through direct (endothelial differentiation) and indirect (release of various angiogenic factors) mechanisms. Stem/progenitor cells can also produce many other factors (e.g., EGF and KGF) that increase the proliferation of keratinocytes, epithelial cells, and myofibroblasts, which are known to be involved in wound healing [14]. Moreover, the implantation of stem/progenitor cells has been demonstrated to improve wound healing in an animal model.

Because c-kit-positive (c-kit^+^) cells in bone marrow can be mobilized into peripheral blood in response to ischemia, inflammation, and injuries including surgical injury [15], [16], we investigated whether surgical injuries affect wound healing through the mobilization and recruitment of c-kit+ cells in order to understand the relative mechanisms in detail.

## Materials and Methods

### Animals

Male 8- to 10-weeks-old C57BL/6 mice were purchased from Japan SLC, Inc. (Shizuoka, Japan). GFP-transgenic mice (C57BL6/Tg14) were provided by Masaru Okabe (Genome Research Center, Osaka University, Osaka) [17]. All animal procedures were approved by the Institutional Animal Care and Use Committee of Yamaguchi University and conformed to the Guide for the Care and Use of Laboratory Animals published by the US National Institutes of Health (NIH Publication No. 85-23, revised 1996).

### Left pneumonectomy

Mice were subjected to surgical injury by left pneumonectomy (Surgery group). Briefly, after the induction of general anesthesia and tracheal intubation, the animals were ventilated with room air at a tidal volume of 10 mL/kg and a rate of 100 strokes/min (Harvard rodent ventilator, model 683, Harvard Apparatus Inc., South Natick, MA) [18]. A left thoracotomy was performed through the fourth intercostal space [19], and the left lung was resected after the ligation of the hilum of the left lung with 6-0 silk [20], [21]. The sham operation was performed by simple incisions of the skin and muscles at the left thoracic wall without a thoracotomy (Sham group).

### Monitoring the release of cytokines and the mobilization of bone marrow stem cells

The mice were killed, and blood samples (about 0.7 ml) were collected from inferior vena cava at 6 and 24 h after surgery (n = 3 to 6 mice at each time point). Nucleated cells were isolated from the peripheral blood by gradient centrifugation [22]. We could got about 5×10^5^ nucleated cells from each mouse. Plasma was also collected and frozen for use.

The concentrations of interleukin-6 (IL-6), tumor necrosis factor alpha (TNF-α), and stromal cell-derived factor 1 (SDF-1) in plasma were measured with mouse ELISA kits (R&D Systems, Inc., Minneapolis, Minnesota) according to the manufacturer's instructions.

The mobilization of stem/progenitor cells were identified by measuring c-kit^+^ and CD34-positive (CD34^+^) cells in peripheral blood, as described previously [23]. Considering the critical roles of SDF-1/CXCR4 signaling pathways in stem/progenitor cells mobilization and recruitment, we also measured c-kit^+^/CXCR4^+^ cells and CD34^+^/CXCR4^+^ cells in this study. Briefly, freshly isolated nucleated cells were stained with PE-conjugated antibodies against the mouse c-kit and CD34 (eBioscience, San Diego) at 4°C for 30 min. After washing the cells with phosphate-buffered saline (PBS), the cells were stained with FITC-conjugated rat anti-mouse CXCR4 monoclonal antibody (BD Pharmingen, San Diego) at 4°C for 30 min. A respective isotype immunoglobulin was used as a negative control. Quantitative flow cytometry was performed using a FACS Calibur. A total of 20000 gated events was collected based on forward and side scatter, and the percentages of positively-stained cells were calculated with Cell Quest software (Becton Dickinson).

### Establishment of wound healing model in GFP^+^ bone marrow-transplanted chimera mice

C57BL/6 mice were subjected to lethal irradiation (10 Gy), and then, 5×10^6^ bone marrow mononuclear cells from GFP-transgenic mice were infused intravenously [23]. GFP^+^ bone marrow-transplanted chimera mice were used for the following wound healing studies 8 weeks later, and >99% of the peripheral blood cells were GFP-positive.

A wound healing model was established in these chimera mice as described previously [24]. Wounds were induced on the dorsal region using a 3-mm punch biopsy (derma punch, Maruho, Japan). A layer of skin was removed to expose the underlying muscle.

### Inhibition of SDF-1/CXCR4 signaling pathway

To evaluate the influence on wound healing of surgical injury and the inhibition of the SDF-1/CXCR4 signaling pathway, mice were selected randomly to undergo either a left pneumonectomy or a sham operation. The mice were further selected randomly to undergo the inhibition of the SDF-1/CXCR4 signaling pathway by an intraperitoneal injection of 10 mg/kg AMD3100 (Sigma, St. Louis, MO), a well-known CXCR4 antagonist [25]–[27]. A control treatment was performed by intraperitoneal injections of PBS. A total of three injections were given 12 h before the operation, immediately following the operation and 12 h after the operation.

### Evaluation of wound healing

Wound healing was recorded every second day with digital photographs. The surface area of wounds was quantitatively measured using Image-Pro Plus software (Media Cybernetics) and was expressed as the percentage of the initial wound size [24].

Mice were sacrificed at 3, 7, or 14 days after surgery, and paraffin sections of skin wound tissue were used for the following histological analyses. The wound diameter indicated the leading edges of the wounded epidermis and was measured in these tissues at 3 and 7 days after surgery [26].

### Histological analysis

The recruitment of c-kit+ cells was estimated by histological analysis, as described previously [27]. Briefly, tissue sections were deparaffinized, after which nonspecific protein blocking with Serum-Free Protein Block (Dako, Carpenteria, CA) was performed for 30 min at room temperature. Sections were then incubated with a PE-conjugated, rat anti-mouse c-kit antibody (eBioscience, San Diego) for 60 min at room temperature. Images were captured from at least 10 randomly selected fields from two separate sections of each sample, using a digital camera (Nikon Digital Sight DS-SMc) with ACT-2U software (Nikon, Japan). The number of bone marrow origin GFP^+^ cells (green) that stained positively for c-kit (red) was also considered to represent c-kit+ cells (merged as yellow color) in wound tissues.

It is generally agreed that wound macrophages play an important role in wound healing. To estimate the recruitment of macrophages, tissue sections were deparaffinized and subjected to antigen heat retrieval. After nonspecific protein blocking as described above, the sections were then incubated with a PE-conjugated, rat anti-mouse F4/80 antibody (eBioscience, San Diego) at 4°C overnight. The mean numbers of macrophages and the bone marrow-derived macrophages (GFP^+^ and F4/80^+^) in each sample were calculated for the statistical analysis.

Wound healing of the skin is a process that involves fibroplasias and angiogenesis. We also observed the differentiation of c-kit+ cells into myofibroblasts and endothelial cells by immunostaining with Cy3-conjugated rat anti-mouse αSMA antibody (Sigma, St. Louis, MO) and PE-conjugated rat anti-mouse CD31 antibody (eBioscience, San Diego), respectively.

### Measurement of the concentrations of growth factors in wound tissues

To further understand the related mechanisms, we measured the concentrations of transforming growth factor beta-1 (TGF-β_1_), vascular endothelial growth factor (VEGF), and epidermal growth factor (EGF) in the wound tissues. Briefly, wound tissues were collected at 3 days after surgery, and then minced and homogenized on ice with the addition of phosphate-buffered saline (PBS). After centrifugation at 4°C, the resulting supernatants were used for ELISA analysis, as described above. The concentrations of TGF-β_1_, VEGF, and EGF were finally corrected by the concentration of total proteins in the supernatants.

### Statistical analysis

All results are presented as the mean ± the standard deviation (SD). Statistical significance was determined by an analysis of variance (ANOVA) followed by the Tukey post hoc test (Dr. SPSS II, Chicago, IL). Differences were considered statistically significant when p<0.05.

## Results

### Surgical injury induced the release of cytokines and the mobilization of stem/progenitor cells

**Figure 1:**
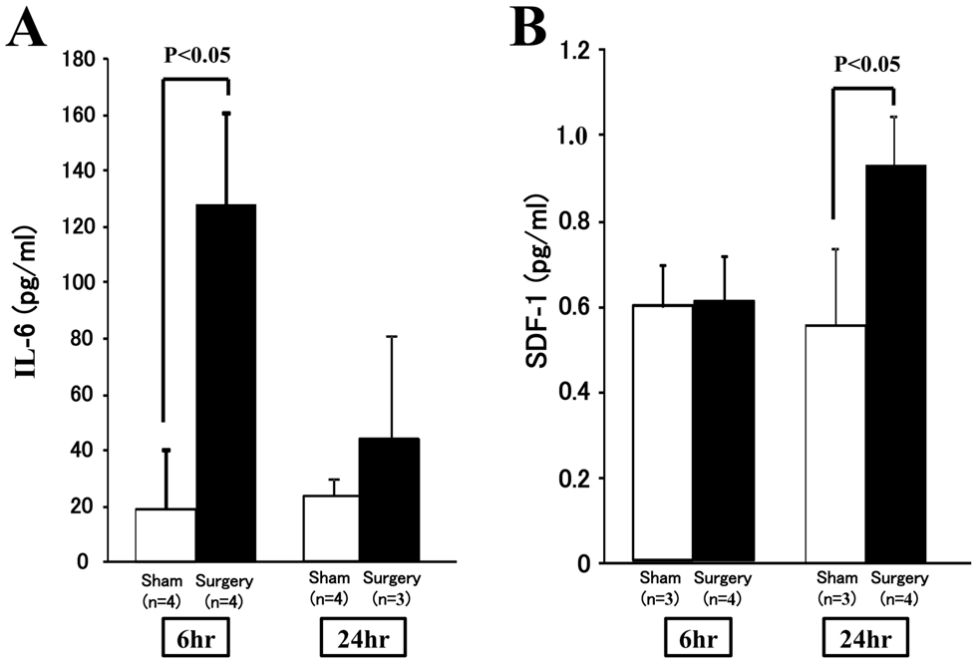
Changes in systemic levels of cytokine IL-6 and chemokine SDF-1 after surgery. **A**) Significant increases in plasma IL-6 concentrations were observed at 6 hr, but not 24 hr after left pneumonectomy. **B**) The concentration of SDF-1 in plasma was also significantly increased at 24 hr, but not 6 hr, after left pneumonectomy.

All surgical processes, including left pneumonectomies, were successfully completed without obvious blood loss (less than 0.1 mL). Several animals with adverse events during surgery, e.g., excessive bleeding, were excluded from the study. Compared with the sham group, the group with pneumonectomies had increased concentrations of the inflammatory cytokine IL-6 in plasma at 6 h, but no increase was observed at 24 h after surgery (*p<0.05*, ***Figure 1A***). The plasma levels of SDF-1, one of the most important chemokines regulating cell migration and mobilization, were also increased 24 h after surgery (*p<0.05*, ***Figure 1B***). TNF-α, however, another popular marker of surgical injury, was not detectable in plasma by ELISA (less than the sensitivity of assay) after pneumonectomy.

**Figure 2:**
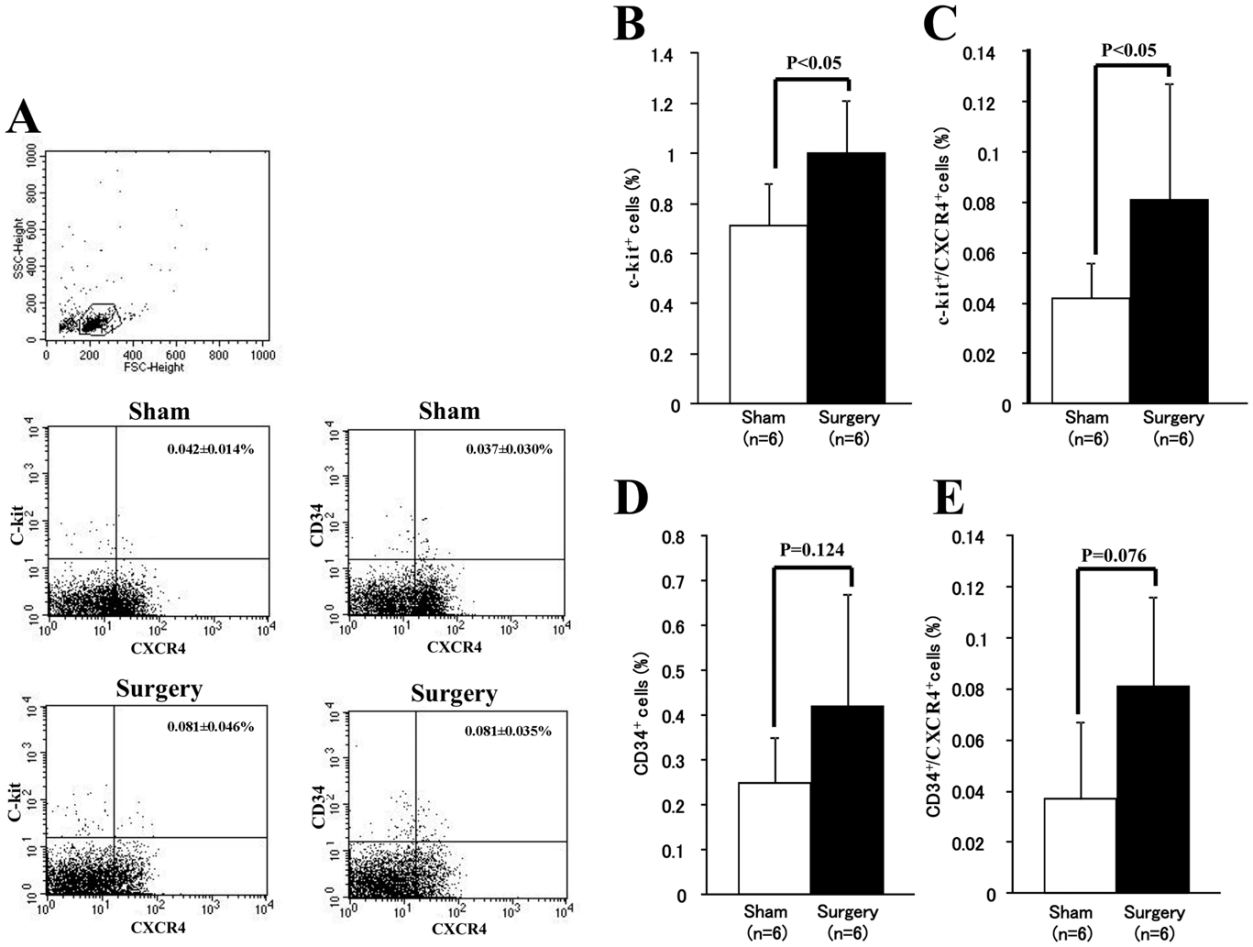
Mobilization of stem/progenitor cells in mice after surgery. Nucleated cells from peripheral blood were used for analysis, and a total of 20000 gated events was collected based on forward and side scatter by using a FACS Calibur. **A.** Representative dot graphs showing the gating area (top dot plot graph) and data of c-kit^+^/CXCR4^+^ cells and CD34^+^/CXCR4^+^ cells for each group. Compared with control mice, quantitative flow cytometry analysis revealed significant increases of c-kit^+^ cells (**B**), c-kit^+^/CXCR4^+^ cells (**C**), CD34^+^ cells (**D**), and CD34^+^/CXCR4^+^ cells (**E**) in the peripheral blood 24 hr after pneumonectomy.

A total of 20000 gated events was collected based on forward and side scatter (upper dot graph, ***Figure 2A***). A quantitative flow cytometry analysis revealed that pneumonectomy induced a significant increase in c-kit^+^ cells (1.0±0.21% *vs.* 0.7±0.17% of Sham; *p<0.05*, ***Figure 2A and 2B***) and c-kit^+^/CXCR4^+^ cells (0.081±0.046% *vs.* 0.042±0.014% of Sham, *p<0.05*; ***Figure 2A and 2C***), as wells as CD34^+^ cells (0.42±0.25% *vs.* 0.25±0.10% of Sham; *p = 0.124*, ***Figure 2D***) and CD34^+^/CXCR4^+^ cells (0.081±0.035% *vs.* 0.037±0.030% of Sham, *p = 0.076*; ***Figure 2A and 2E***) 24 h after surgery. Also, c-kit/CXCR4 double positive cells and CD34/CXCR4 double positive cells, which are likely to be contributing to the wound healing, were significantly increased. These findings indicate that surgical injury increases the release of cytokines/chemokines and that it induces the mobilization of stem/progenitor cells. These results confirmed that the surgical stress is sufficient to induce cytokine secretion and stem/progenitor cells mobilization.

### Surgical injury accelerated wound healing, which was negated by the inhibition of SDF-1/CXCR4 signaling pathway

**Figure 3:**
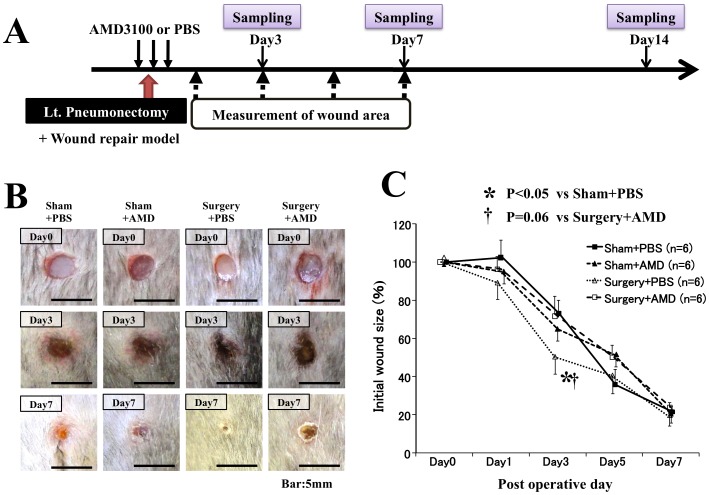
Influence of wound healing by surgical injury and inhibition of SDF-1/CXCR4 signaling pathway. **A**) Timelines depicting the establishment of wound healing model, left pneumonectomy, AMD3100 administrations, and assessments. **B**) Representative pictures of wound healings for mice from each group are shown. **C**) The quantitative analysis showed that the wound areas in the mice that underwent pneumonectomy and PBS injection (Surgery+PBS group) were significantly smaller than those of mice that received sham operation and PBS injections (Control+PBS group), only 3 days after surgery. However, the administration of AMD3100 negated the accelerated wound closure after surgery (Surgery+AMD group) 3 days after surgery but did not affect wound healing in mice without surgery (Control+AMD group). No significant differences were observed among groups at 1, 5, and 7 days after treatment.

**Figure 4:**
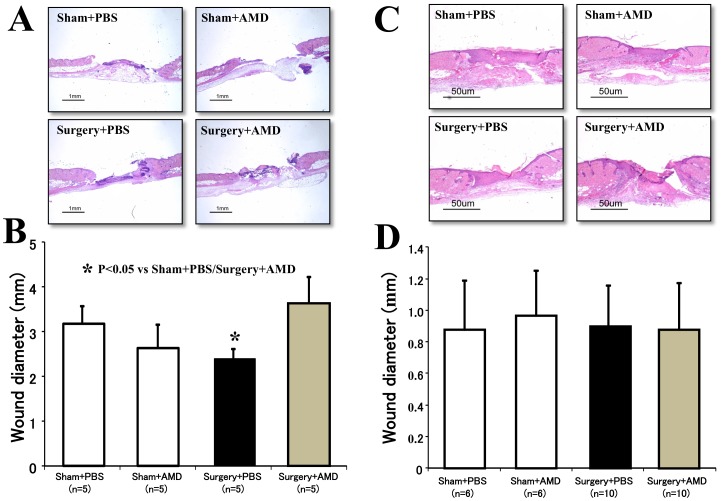
Histological findings of wound tissue. Sections with hematoxylin and eosin staining were used to examine the re-epithelization of wounded skin in mice at 3 days (**A**) and 7 days (**B**) after surgery. A quantitative analysis revealed significantly smaller wound widths in mice that received pneumonectomy (Surgery+PBS group) than mice that received a sham operation (Control+PBS group) 3 days after surgery (**A**). The administration of AMD3100 significantly extended the wound width in mice subjected to pneumonectomy (Surgery+AMD group, p<0.05 vs. Surgery+PBS group) but did not change in mice that received sham operations (Control+PBS group), 3 days after surgery. There was no difference in the wound width among the groups at 7 days after treatment (**B**).

The flowchart summarizing the experiments is shown in ***Figure 3A***. The relative area of skin wound on the dorsa was measured to be significantly smaller in the Surgery+PBS group than in the Sham+PBS group 3 days after surgery (50.3±8.0% *vs.* 73.0±7.0%, *p<0.05*; ***Figure 3C***). Similarly, the wound diameter in the Surgery+PBS group was also significantly smaller than that in the Sham+PBS group 3 days after surgery (***Figure 4A***). However, neither the wound area nor the wound diameter differed between the groups at other time points after surgery (***Figure 3C*** ***,*** ***4B***). These findings indicate that surgical injury accelerates wound healing in the early phase.

Inhibition of the SDF-1/CXCR4 signaling pathway by AMD3100 suppressed any postoperative acceleration of the wound healing 3 days after surgery (***Figure 3C*** ***,*** ***4A***). The relative area of the skin wounds did not differ, however, among the Sham+PBS, Sham+AMD, and Surgery+AMD groups, indicating that neither PBS injection nor AMD3100 administration affected wound healing.

### Inhibition of SDF-1/CXCR4 signaling pathway suppressed the recruitment of c-kit+ cells into wound tissue after surgery

**Figure 5:**
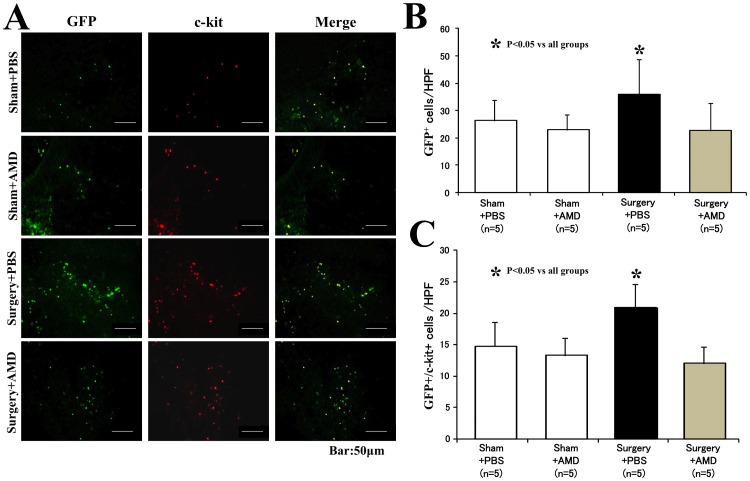
Recruitment of c-kit+ cells into wound tissues. **A**) Representative images of the recruitment of c-kit+ cells into the wound tissue are shown for each group. Bone marrow-derived cells (GFP^+^) appeared as green fluorescence (left panel), and c-kit+ cells were detected as red (middle panel). Some GFP^+^ cells were stained positively for c-kit (merged as yellow color, right panel). **B**) A quantitative analysis revealed that the number of total bone marrow-derived cells (GFP^+^) within wound tissue was significantly greater in the Surgery+PBS group than the Control+PBS group but that the number of total bone marrow-derived cells was less in the Surgery+AMD group than in the Surgery+PBS group. **C**) The number of c-kit+ cells (GFP^+^/c-kit^+^) was also significantly greater in the Surgery+PBS group than the Control+PBS group but less in the Surgery+AMD group than the Surgery+PBS group.

Many GFP^+^ bone marrow-derived cells (green) were detected in wound tissues (***Figure 5A***). A quantitative analysis showed that the number of GFP^+^ cells in wound tissue was significantly greater in the Surgery+PBS group than that in the Sham+PBS group (35.9±12.8/HPF *vs.* 26.3±7.6/HPF, *p<0.05*; ***Figure 5B***). The administration of AMD3100 decreased the number of GFP^+^ cells to a remarkable degree in the mice subjected to surgical injury (22.6±10.1/HPF of Surgery+AMD group, *p<0.05 vs.* Surgery+PBS group) but did not change in the mice that received sham operations (23.0±5.4/HPF of Sham+AMD group, *p = 0.21 vs.* Sham+PBS). These results suggest that surgical injury induced the recruitment of bone marrow-derived cells into wound tissue, which was suppressed by the intervention of the SDF-1/CXCR4 signaling pathway.

**Figure 6:**
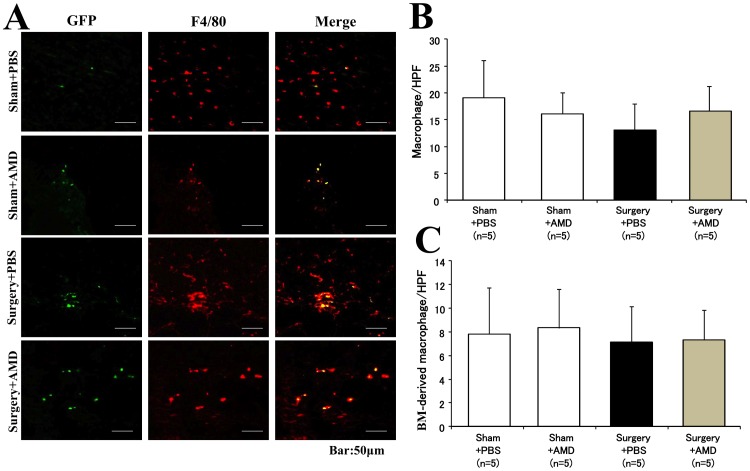
Recruitment of bone marrow-derived macrophages into wound tissues. **A**) Representative images in each group are shown. Bone marrow-derived cells (GFP^+^) in wound tissue appeared as green fluorescence (left panel), and macrophages were detected as red fluorescence by immunostaining for F4/80 (middle panel). Some GFP^+^ cells were stained positively for F4/80 (merged as yellow color, right panel). A quantitative analysis revealed that neither the total number macrophages (**B**) nor the bone marrow-derived macrophages (**C**) within wound tissue differed significantly among the groups.

An immunostaining analysis was used to further characterize these recruited bone marrow-derived cells. The GFP^+^/c-kit^+^ cells (yellow, ***Figure 5A***), a subpopulation of c-kit+ cells of bone marrow origin, was significantly greater in the Surgery+PBS group than in the Sham+PBS group (20.9±3.8/HPF *vs.* 14.8±3.8/HPF, *p<0.05*; ***Figure 5C***). However, the number of c-kit+ cells was decreased in the Surgery+AMD group, in which the mice received AMD3100 after pneumonectomy (12.0±2.7/HPF, *p<0.05 vs.* Surgery+PBS group; ***Figure 5C***). We also quantified the F4/80^+^ macrophages in wound tissue (***Figure 6A***). Neither the total number of macrophages (***Figure 6B***) nor the bone marrow-derived macrophages (***Figure 6C***) differed significantly among the groups. These results suggest that the recruitment of stem cells, but not macrophages, contribute to accelerate wound healing after surgical injury.

### Differentiations and of c-kit+ cells

**Figure 7:**
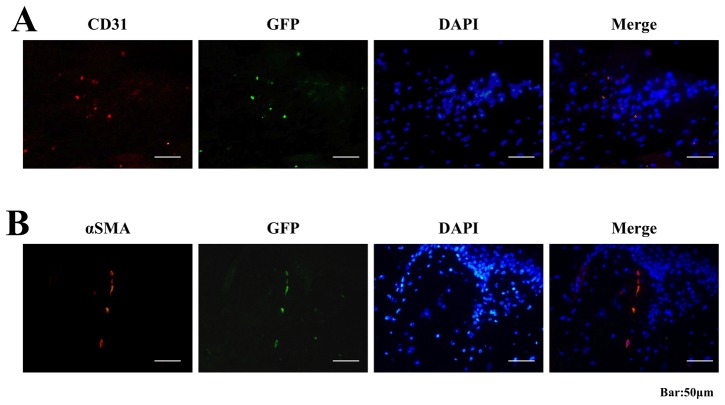
Differentiations of c-kit+ cells in wound tissues. An immunostaining analysis revealed that a few of the GFP^+^ cells in the wound tissue stained positively with the endothelial specific marker of CD31 (**A**) and myofibroblast specific marker of αSMA (**B**) 2 weeks after surgery, indicated the differentiations of c-kit+ cells into endothelial cells and myofibroblasts.

Although we did not quantify the number, some GFP^+^ cells in granulation tissue were stained positively for αSMA and CD31 (yellow, ***Figure 7***). This finding indicates that the recruited c-kit+ cells could differentiate into myofibroblasts and endothelial cells for direct participation in wound healing.

### Paracrine factors in wound tissues

**Figure 8:**
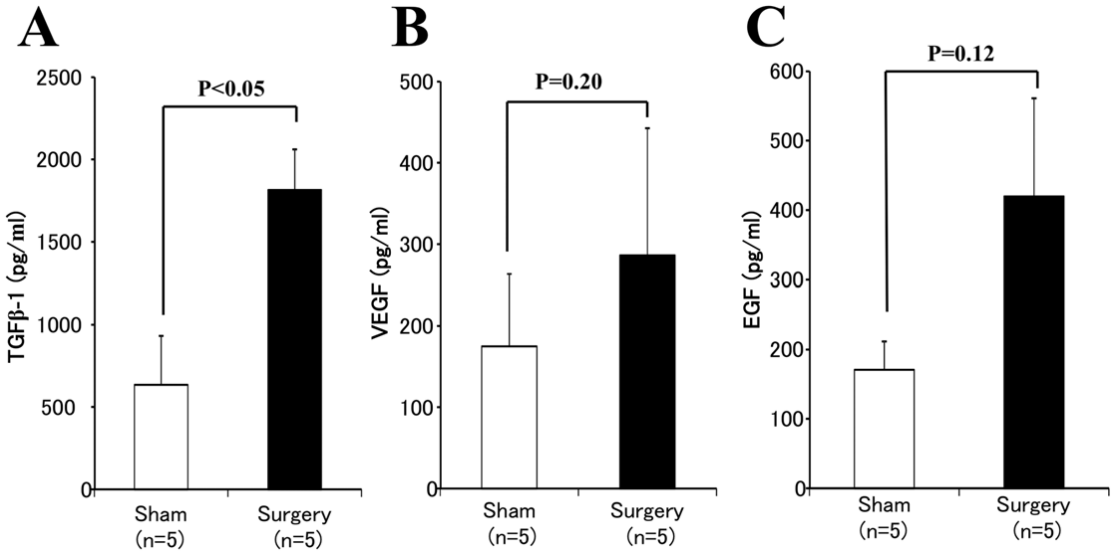
The levels of TGFβ-1, VEGF, and EGF in wound tissue. The level of TGF-β_1_ (**A**) in the wound tissue was significantly higher in surgery group than sham group, but the levels of VEGF (**B**) and EGF (**C**) in the wound tissue did not differ significantly between groups, at 3 days after surgery. Data represents 5 separated experiments with duplicated assays.

Compared with the sham group, the group with pneumonectomies had significantly increased the concentration of TGF-β_1_, the important cytokine of wound healing, in the wound tissue at 3 days (*p<0.05*, ***Figure 8A***). VEGF and EGF, two of critical growth factors of wound healing, were also measured at higher levels in the surgery group than sham group, but it was not significant because of a big variation within the limited sample size (***Figure 8B and C***).

## Discussion

It is well known that hypoxia and ischemia can stimulate the release of various factors, such as HIF-1, VEGF, and SDF-1α, to induce the mobilization and recruitment of stem/progenitor cells [29]–[31]. Several recent investigations have also reported that surgical injuries increase the number of stem/progenitor cells in peripheral blood [6], [7]. In this study, we found that pneumonectomy increased IL-6 and SDF-1α, as well as the c-kit- or CD34- positive cells in peripheral blood, and our findings confirm that surgical injury can induce the release of cytokines/chemokines and the mobilization of stem/progenitor cells. Sham operation in this study was performed by only skin and muscle incision, because the purpose of this study was to investigate how surgical injury affects wound healing. Thoracotomy and pneumonectomy are generally considered as surgical injuries.

Although direct implantation of bone marrow-derived mesenchymal stem cells was found to improve wound healing, the role of mobilized stem/progenitor cells in wound healing after surgery is still unclear. Using an established wound healing model in GFP^+^ bone marrow transplanted chimera mice, we found that surgical injury caused by pneumonectomy increased the number of GFP^+^ bone marrow-derived stem cells in wound tissues and that it accelerated early wound healing. However, either the increased accumulation of bone marrow-derived cells in wound tissue or the accelerated wound healing in mice after left pneumonectomy were completely suppressed by administering AMD3100, a well-known antagonist of SDF-1/CXCR4 signaling pathways [28], [29]. These results clearly demonstrated that the mobilized c-kit+ cells after surgery could recruit into the wound tissue to accelerate healing.

Previous studies have reported that stem/progenitor cells can repair skin wounds by inducing angiogenesis, differentiating into endothelial cells and myofibroblasts to directly regenerate new vessel and granulation tissue. However, the mechanisms of stem/progenitor cells for wound healing after surgery remain unclear. We have tried to understand the mechanisms of accelerated wound healing after surgical injury by a histological analysis. Pneumonectomy significantly increased the recruitment of c-kit+ cells but did not change the number of F4/80-positive macrophages. It is generally accepted that wound macrophages play a key role in cutaneous healing. However, this suggested that the mobilization and recruitment of c-kit+ cells, but not macrophages, were critical for accelerating wound healing. Wound healing of the skin represents a dynamic process involving fibroplasia and angiogenesis. Furthermore, we observed that some c-kit+ cells were differentiated into endothelial cells and myofibroblasts within the granulation tissue, to directly participate in wound healing.

Although c-kit+ cells have been demonstrated to differentiate into endothelial and keratinocytes, these differentiations was observed rarely and we thought that the directly differentiation of played minor role on wound healing after surgery. A number of previous studies have demonstrated that stem/progenitor cells can produce various growth factors to improve wound healing, that was well-known as the paracrine mechanisms [14]. Therefore, we measured the concentrations of several critical growth factors, including TGF-β_1_, VEGF, and EGF in the wound tissue. All of these factors were increased by surgery, although two of them were not significant. Although further experiments are required to understand the complex mechanisms on stem cells working for wound healing, our data indicated that the mobilization and recruitment of stem/progenitor cells after surgical injury contributed to accelerate wound healing through at least partly the paracrine mechanisms.

We thought that the mobilization of c-kit+ cells is just one of the mechanisms of the accelerated wound healing after surgery. Surgical injury is known to increase may at least partially affect the wound healing. In this study, we found that the disruption of SDF-1/CXCR4 signaling pathways by AMD3100 was able to reduce the recruitment of c-kit+ cells into wound tissue and to attenuate the accelerated wound healing. However, these findings were based on a mild surgical injury model of pneumonectomy. Further experiments using different surgical injuries and wound healing models, including skin incision or skin flap models are required to figure out how surgical injury affects wound healing.

Many recent studies have demonstrated that either the number or the function of c-kit+ cells were decreased in patients with advanced age and several complications (e.g., diabetes mellitus) [30], [31]. Accordingly, delayed wound healing is usually found in those patients. Further studies are required to demonstrate the causal relationship between the impaired mobilization and recruitment of c-kit+ cells and the delay of wound healing after surgery in patients with advanced age and diabetes. It would be interesting to know whether the number and quality of c-kit+ cells in peripheral blood could be a potential biomarker that predicts the risk of delayed wound healing in patients after surgery.

In conclusion, we found that surgical injury can induce the mobilization of c-kit+ cells. These mobilized c-kit+ cells would be recruited into the wound tissue to accelerate early wound healing after surgery. Our findings suggest that c-kit+ cells will be a novel and effective target in wound healing after surgery.
